# Black soybean tempeh and purple sweet potato improve sperm quality in streptozotocin-induced diabetic rats

**DOI:** 10.14202/vetworld.2020.2534-2540

**Published:** 2020-11-27

**Authors:** Abdul Gofur, Agung Witjoro, Siti Nur Arifah, Mochammad Fitri Atho’illah, Yuslinda Annisa, Sri Rahayu Lestari

**Affiliations:** 1 Department of Biology, Faculty of Mathematics and Science, Universitas Negeri Malang, Jalan Semarang no. 5, 65145, Malang, East Java, Indonesia; 2 Department of Biology, Faculty of Mathematics and Natural Sciences, Brawijaya University, Malang 65145, East Java, Indonesia

**Keywords:** antioxidant, black soybean tempeh, purple sweet potato, spermatozoa

## Abstract

**Background and Aim:**

Hyperglycemia increases advanced glycation end-product (AGE) production, and the activity of receptor for AGE (RAGE) in testis, which leads to testicular histopathological damage and infertility. This research investigated the effect of black soybean tempeh (BST), purple sweet potato (PSP), and its combination on AGE and RAGE expression and spermatozoa quality in streptozotocin (STZ)-induced diabetic rats.

**Materials and Methods:**

The rats were given high-fat diets for 5 weeks, then were injected intraperitoneally with multiple low doses of STZ (30 mg/kg body weight). Diabetes mellitus (DM) rats were divided into seven groups: DM, DM+glibenclamide, DM+BST, DM+PSP, and DM+combination of BST and PSP in ratio 1:3, 2:2, and 3:1 as C1, C2, and C3, respectively. The rats were treated for 30 days. Testicular AGE and RAGE expression and spermatozoa quality were measured.

**Results:**

The combination of BST and PSP significantly decreased AGE and RAGE expression in testicular organs and improved spermatozoa quality compared to the normal group.

**Conclusion:**

The combination of BST and PSP can be used as future alternatives to improve spermatozoa quality in DM patients.

## Introduction

Diabetes mellitus (DM) is a chronic metabolic disease that causes morbidity and mortality on a large scale. DM is characterized by chronic hyperglycemia when carbohydrates, lipids, and proteins are consumed, which is caused by metabolism malfunction due to insulin damage. DM induces various metabolic syndromes and causes degenerative function in organs, such as in the testis [1-3]. It also plays a role in infertility, especially Type 2 DM (T2DM) in males, which is common in children, adolescents, and male adults of reproductive age [4]. DM causes histopathological damage in the testis and contributes to male infertility by triggering abnormal spermatogenesis, changes in sperm quality and/or function, and decreases in sperm motility, hormone deregulation, and sexual disorder [5].

During the development of diabetes, the balancing between reactive oxygen species (ROS) production and antioxidants is disturbed. Excess ROS levels can damage cell structures, including protein components and DNA, which cause damage to cellular functions. In diabetics, oxidative stress causes DNA damage in the testis, thereby disturbing spermatogenesis function and causing infertility in males [6]. In hyperglycemia, advanced glycation end-product (AGE) is a free radical product that can increase toxicity levels and has the potential to cause systemic damage [7]. AGE damages macromolecules by interacting with receptor for AGE (RAGE). The binding of AGE to RAGE activates several signaling pathways, produces oxidative stress, and increases membrane protein expression and cytokine production. RAGE activities can increase ROS production, which causes DNA fragmentation [8,9]. The inhibition of AGE formation and the binding of AGE to RAGE may suppress pathogenicity and complications in T2DM.

Antioxidants play an essential role in degenerative diseases related to cell and tissue oxidation by ROS [10]. Phenolic compounds in plants have antioxidant properties and display radical scavenger activity. Black soybean (*Glycine max* [L.] Merrill.) and its derivative products, such as tempeh, contain various phenolic compounds with antioxidant abilities [11,12]. A previous study suggested that the addition of microorganisms to soybeans can change the composition of its phenolic compounds and increase their beneficial functions [13]. Thus, fermented soybeans (e.g., tempeh) possess better antioxidant, physicochemical, and nutritive properties than unfermented soybeans [14]. Purple sweet potato (PSP) (*Ipomoea batatas* L.; PSP) contains high anthocyanin and antioxidant properties that can improve blood and various organ damages caused by oxidative stress [15,16]. Our previous study suggested that non-fermented black soybean improves sperm quality in DM rats when combined with PSP [17]; however, limited information is available regarding the impact of black soybean tempeh (BST) on the sperm quality of T2DM individuals when combined with PSP. The antioxidant contents in both BST and PSP are expected to be therapeutic agents that can be used in DM treatments.

This research investigated the effect of BST, PSP, and its combination on AGE and RAGE expression and spermatozoa quality in streptozotocin (STZ)-induced diabetic rats.

## Materials and Methods

### Ethical approval

All research procedures had been approved by the Research Ethics Commission of Brawijaya University, Malang (No. App. 878-KEP-UB).

### Study period and location

This study was conducted between June and October 2019 at Animal House, Department of Biology, Faculty of Mathematics and Science, Universitas Negeri Malang.

### PSP preparations

Three kg PSP was purchased from a traditional market near Kawi Mountain, Malang Regency. The PSP was cleaned and cut 3-4 mm thick, then put in the oven at 40-50°C approximately for3-4 days until it became completely dried. The dried PSP was then ground into flour.

### BST preparations

Black soybeans var. Detam-1 was purchased from BALITKABI, Malang Regency. The black soybeans were cleaned, washed, and soaked in water for 12-18 h. Then, the peel was removed, and the soybeans were washed and rinsed using clean water. Next, the soybeans were boiled until the seeds were soft, and then aerated until warm. Tempeh yeast was sprinkled on the soybeans, which were stirred evenly (1.5 g tempeh yeast for 2 kg soybeans). The soybeans were packaged in 1-2 cm thick plastic and kept at room temperature for 1-2 days until the entire soybean surface was covered with fungus. The resulting tempeh was sliced thinly and dried in an incubator at 60°C for 2-3 days. Dried BST was then ground into flour.

### Extraction of BST and PSP

BST and PSP (10 g each) were diluted in 100 mL of distilled water. The solution was stirred for 30 min, and then centrifuged at 3000 rpm for 15 min. The supernatant was removed with a micropipette and kept in the dark glass bottle at 4°C.

### Animal model and experimental design

Thirty-twoeight-weeks-old male Wistar rats (*Rattus norvegicus*) of 150±10 g weight were housed individually in standard cages and given free access to food and water. The rats had an acclimatization period of 7 days. The animals were then divided into normal and treatment groups. The normal group was given a normal diet, while the control and treatment groups were given a high-fat diet (HFD) and 10% sucrose drinking water for 5 weeks. The HFD contained 8.84% carbohydrates, 8.5% protein, and 34.2% fat, with the remaining contents consisting of fiber, minerals, and vitamins [18]. HFD-fed rats were injected intraperitoneally with STZ in multiple, low doses (30 mg/kg body weight [BW], diluted in citrate-buffered saline pH 4.5). Blood glucose levels were checked before and after STZ injection. Twenty-eightrats were considered to have DM when their fasting blood glucose level was >200 mg/dL [12].

The DM indicated rats were further divided into seven groups with four rats for each group: DM, DM treated with glibenclamide (0.6 mg/kg BW), DM treated with BST, DM treated with PSP, and DM treated with a combination of BST and PSP in 1:3, 2:2, and 3:1 ratios (C1, C2, and C3, respectively). Treatments were given for 30 days [17]. The long period of treatment was chosen asspermatogonia and spermatocytes in the seminiferous tubules has effect over agent exposure during spermatogenesis stages on 28+3 days [19]. Thus we evaluated for sperm quality after STZ exposure. After treatments, rats were then euthanized using isoflurane (4%).

### Measurement of testicular index

The testicular organs were removed and washed in triplicate using phosphate-buffered saline (PBS). Testis was weighed and measured for testicular index calculations, according to Equation (1) [20]:

Testis index=(Testis weight/Body weight)×100(1)

### Measurement of sperm concentration and motility

The cauda epididymis was removed and placed into a Petri dish containing 1 mL NaCl 0.9%, then minced using a scalpel. The sperm was measured under a light microscope (Olympus, Japan) with 400×. The sperm count results were reported as the number of counted sperm/mL. The spermatozoa concentration was analyzed using the following Equation (2):

Spermatozoa concentration=(Number of spermatozoa/5)×10^6^(2)

The motility was determined by calculating the number of spermatozoa that either moved or did not move per 100 spermatozoa in 10 visual fields under a light microscope with 100×. The motility of spermatozoa is given as the percentage of motile sperms relative to the total counted sperms [17].

### Measurement of testosterone level

The serum testosterone levels were measured using an enzyme-linked immunosorbent assay (ELISA) at 450 nm wavelength, following the protocol provided by the ELISA assay kit (E-EL-0072: Elabscience, USA).

### Immunohistochemical (IHC) assay

The AGE and RAGE expressions in the testis were measured using an IHC assay.The slides of testis were stained using rat anti-AGE primary antibodies (Cat. No. SC 365154, Santa Cruz Biotechnology, USA), mouse anti-RAGE primary antibodies (Cat. No. SC-65154, Santa Cruz Biotechnology, USA), goat anti-rat IgG fluorescein isothiocyanate (FITC) secondary antibodies (Cat. No. 02-16-06, KPL, USA), goat anti-mouse IgG tetramethyl-rhodamine isothiocyanate secondary antibodies (Cat. No. Ab6768, Abcam, USA), and BSA 2% in a ratio of 1:1:1500 and incubated for 1 h. The slides were washed using PBS pH 7.4 in triplicate, then were dried and mounted using Canada balsam. The intensity of each slide was observed under a fluorescence microscope (FSX 100, Olympus, Japan).

### Statistical analysis

The data were analyzed using one-way ANOVA (p<0.05). All data are given as the average±standard deviation. The significant difference between the groups was further tested with the *post hoc* test, using the Duncan multiple range test.

## Results

### Combination of BST and PSP decreases BW and glucose in DM rats

Increased final BW in the rats corresponded to increases in blood glucose levels in the DM groups (Table-1). The BST, PSP, and combination treatment groups had decreased final BWs, and lowered blood glucose levels compared to the DM group.

**Table-1:** Weight and blood glucose levels in normal and treatment groups.

Groups	Initial weight (g)	Final weight (g)	Initial glucose (mg/dL)	Final glucose (mg/dL)
Normal	169.00^a^±9.64	191.00^b^±6.56	116.33^a^±1.53	122.67^c^±2.52
DM	172.67^a,b^±11.59	214.33^c^±4.93	247.00^b^±8.54	422.00^d^±8.72
DM+Glb	173.67^a,b^±10.21	166.33^a^±5.51	260.33^c^±6.11	76.33^a^±3.06
DM+BST	174.33^a,b^±12.50	159.67^a^±7.09	321.67^d^±6.51	80.33^a^±2.52
DM+PSP	190.00^b,c^±9.17	158.33^a^±5.51	385.67^e^±4.04	75.33^a^±25.52
DM+C1	179.00^a,b^±12.12	159.00^a^±3.61	391.33^e^±7.02	111.67^b^±10.41
DM+C2	177.33^a,b^±2.51	163.33^a^±4.16	436.00^g^±7.94	106.67^b^±5.03
DM+C3	201.00^c^±2.65	165.33^a^±5.03	420.00^f^±7.00	108.67^b^±6.51

Data are displayed as mean±SD. Different letters indicate a significantly difference (p**<**0.05) between each group based on DMRT *post hoc* test. Normal=Healthy rats, DM=STZ+HFD only, DM+Glb=DM+Glibenclamide 0.6 mg/kg BW, DM+BST=DM+Black soybean tempeh, DM+PSP=DM+Purple sweet potato, DM+C1=DM+Combination of BST and PSP with ratio 1:3, DM+C2=DM+Combination of BST and PSP with ratio 1:1, DM+C3=DM+Combination of BST and PSP 3:1. SD=Standard deviation, DMRT=Duncan multiple range test, DM=Diabetes mellitus, STZ=Streptozotocin, HFD=High-fat diet

### Combination of BST and PSP improves the sperm quality on DM rats

No significant differences in testicular weights were found between groups. The testicular index in the treatment groups was significantly different (p*<*0.05) compared to the DM group (Table-2). Sperm concentrations and sperm motility significantly increased (p<0.05) in the DM treatment groups compared to the DM group. The combination of BST and PSP restored sperm concentrations and sperm motility in the DM rats (Table-2).

**Table-2:** Testicular weight, testicular index, sperm concentration, motility, and testosterone levels in normal and experimental groups.

Groups	Testicular weight (g)	Testicular index	Sperm concentration (×10^6^)	Motility (%)	Testosterone levels (pg/dL)
Normal	0.99±0.18	5.19^b,c^±1.13	76.37^c^±5.39	79.25^c^±10.99	2.11^b,c^±0.79
DM	0.64±0.31	2.94^a^±1.37	31.63^a^±2.29	42.56^a^±3.80	0.52^a^±0.13
DM+Glb	0.92±0.24	5.55^b,c^±1.65	71.33^b,c^±8.47	71.84^b,c^±8.97	3.11^c,d^±0.38
DM+BST	1.01±0.17	6.32^b,c^±0.89	63.70^b^±5.57	61.01^b^±9.41	5.19^f^±1.72
DM+PSP	0.39±0.48	3.62^a,b^±2.28	38.47^a^±7.68	48.07^a^±5.76	1.84^a,b^±0.54
DM+C1	0.67±0.12	4.21^a,b^±0.79	65.87^b^±4.44	65.85^b^±3.28	4.75^e,f^±0.58
DM+C2	1.24±0.28	7.57^c^±1.77	67.99^b,c^±2.48	69.64^b,c^±4.14	3.51^d,e^±0.06
DM+C3	0.99±0.35	6.00^b,c^±2.06	64.82^b^±2.76	70.41^b,c^±2.28	3.85^e,f^±1.30

Data are displayed as mean±SD. Different letters (^a-f^) indicate significantly difference (p<0.05) between treatment groups based on DMRT *post hoc* test. Normal=Healthy rats, DM=STZ+HFD only, DM+Glb=DM+Glibenclamide 0.6 mg/kg BW, DM+BST=DM+Black soybean tempeh, DM+PSP=DM+Purple sweet potato, DM+C1=DM+Combination of BST and PSP with ratio 1:3, DM+C2=DM+Combination of BST and PSP with ratio 1:1, DM+C3=DM+Combination of BST and PSP 3:1. SD=Standard deviation, DMRT=Duncan multiple range test, DM=Diabetes mellitus, STZ=Streptozotocin, HFD=High-fat diet

The DM group without treatment had the lowest level of testosterone in their blood serum. The level of testosterone increased significantly (p<0.05) in the DM treatment groups compared to the DM group without treatment (Table-2).

### Combination of BST and PSP ameliorates AGE and RAGE expression on testicular organ

**Figure-1:**
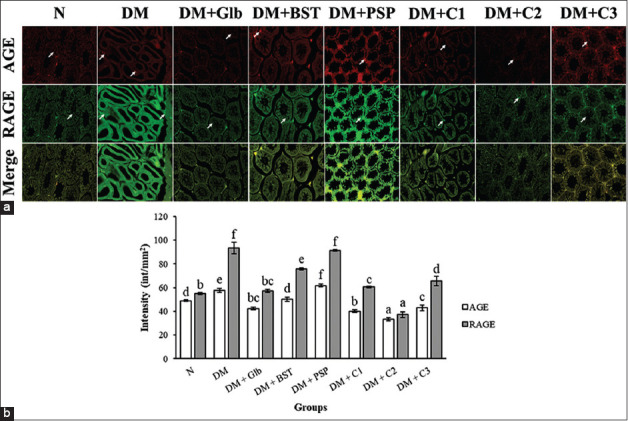
Advanced glycation end-product (AGE) and receptor for AGE (RAGE) expression in normal and treatment groups. (a) The immunohistochemical double staining of the testes in diabetes mellitus (DM) rat model with 400×. AGE=red (rhodamine); RAGE=green (fluorescein isothiocyanate). White arrow pointed the AGE or RAGE expression. (b) The combination of black soybean tempeh (BST) and purple sweet potato (PSP) reduces AGE and RAGE expression in DM rat model. The bar displayed as mean±standard deviation. The different letters indicated a significantly difference (p<0.05) compared between each group based on Duncan multiple range test. Normal=Healthy rats, DM=Streptozotocin+High-fat diet only, DM+Glb=DM+Glibenclamide 0.6 mg/kg BW, DM+BST=DM+Black soybean tempeh, DM+PSP=DM+Purple sweet potato, DM+C1=DM+Combination of BST and PSP with ratio 1:3, DM+C2=DM+Combination of BST and PSP with ratio 1:1, DM+C3=DM+Combination of BST and PSP 3:1.

The IHC assay indicated changes and damages to the seminiferous tubules in the testis of each treatment group compared to the normal group. The intensity of AGE and RAGE expression in the DM rats treated with a combination of BST and PSP at a 1:1 ratio decreased significantly (p<0.05) compared to the DM group without treatment (Figure-1).

## Discussion

T2DM is a chronic metabolic disease related to hyperglycemia and insulin resistance. Glucose metabolism abnormalities and oxidative stress are the main cause of several diabetes complications, such as vascular diseases, kidney damage, and reproduction dysfunction [21]. In this study, the application of HFD and STZ injections in multiple low doses was used to induce T2DM in male rats. HFD/STZ models involved the combination of HFD and sucrose that caused hyperinsulinemia, insulin resistance, and glucose intolerance that caused pancreatic-β cells damage when followed by the STZ injections [22]. Both stressors were designed to imitate T2DM pathology in a shorter time period than in humans [23]. The results indicated that HFD-fed and STZ-induced rats had increased body weights and higher blood glucose levels.

The increased blood glucose levels were caused by a dysfunction in the transportation of glucose transport into the muscle, adipocytes, and peripheral tissues, which increased glycogen damage, the gluconeogenesis process, and hepatic glucose production [24]. STZ-induced diabetic significantly increased oxidative stress during the initial development of DM, and contributed to testicular dysfunction, resulting in decreased fertility potential caused by steroidogenesis and spermatogenesis disorder. STZ-induced diabetic lead to testicular dysfunction by decreasing Leydig cell functions and testosterone production due to the non-existence of insulin stimulation in Leydig cells and significant decrease in testosterone, follicle-stimulating hormone and luteinizing hormone serum levels. Hyperglycemia was involved in sperm concentration and motility, steroidogenesis changes, and caudal epididymal disorder, which inhibited sperm maturation [25].

The effect of T2DM on male reproductive function is caused by an imbalance between ROS and antioxidants in spermatozoa in the seminiferous tubules, DNA fragmentation of sperm mitochondria, and sperm DNA damage that change sperm parameters and induce infertility in male individuals [26]. Excess ROS production has an impact on AGE formation. AGE is a product of glycoside through a non-enzymatic reaction between glucose and protein amino groups, lipids, and DNA under hyperglycemic conditions. AGE changes the normal function of macromolecules directly by producing ROS, independently or indirectly, through the activation of RAGE. RAGE is the receptor of ligand binding that increases cellular dysfunction in inflammatory disorders during DM development [27].

The IHC results indicated a significant increase in AGE and RAGE expression in the testicular organs of the DM group compared to the normal group. RAGE is expressed in low levels in normal tissues; however, there was an increase in RAGE expression in the testis, epididymis, and spermatozoa in DM individuals, which caused tissue damage [28]. The significant increases in AGE and RAGE in T2DM pathophysiology can be mitigated with exogenous antioxidants that are found in some plants.

The results showed that a combination of BST and PSP (1:1) suppressed AGE formation and RAGE activation significantly in testis of DM model rats. Black soybeans have polyphenolic antioxidant compounds, such as isoflavones and phenolic acids, which have the ability to protect against free radicals. Antioxidant contents in black soybeans can be improved through fermentation [29]. The fermentation process of turning soybeans into tempeh modifies the active isoflavone compounds, which causes an increase in antioxidant capacities in tempeh. During the fermentation process, partial divisions or changes in glucoside occur, along with an increase in glucoside and glucuronidase activities, which release strong antioxidant compounds formed through flavonoid transformations [30,31]. PSP also has a high antioxidant content and contains anthocyanin color pigments that are useful antioxidants, with higher stability compared to other anthocyanin sources [32,33]. Antioxidant contents in BST and PSP become the main factor in the improvement of testicular dysfunction and sperm parameters, as well as the AGE and RAGE formation in the testis of DM model rats.

Polyphenol compound groups have aromatic rings with a hydroxyl group, which makes them a strong antioxidant that could protect cells from oxidative damage and limit the risk of various degenerative diseases related to oxidative stress, including diabetes. Antioxidants also play a role in protecting spermatozoa from damage due to ROS oxidation [34,35]. Based on the results in Table-2, sperm concentrations and motility were significantly increased in DM model rats treated with a combination of BST and PSP. In addition, to prevent damage in spermatozoa, anthocyanin compound contents in PSP could improve damage and protect pancreatic-β cells from glucose-induced oxidative stress in T2DM patients [12,36].

The antioxidant abilities of a compound depend on its capacity to donate electrons or hydrogen atoms to free radical molecules. Polyphenol compound groups, such as flavonoids, isoflavones, and anthocyanin, contain one or more aromatic rings with one hydroxyl group or more (C-OH) and other substitutions. Antioxidant activities in polyphenol structures are caused by three key positions in the polyphenol compound structure located on the ortho-3′,4′-dihydroxy in ring B, 2,3-double conjunction 4-oxo group in ring C, 3-OH group in ring C, and 5-OH group in ring A. The structure plays an essential role in antioxidant activity as a free radical scavenger and in the antiglycation in the inhibition of AGE formation inhibition [37-39].

Antiglycation activity by antioxidants can occur through the inhibition of the three main pathways of AGE formation, namely, the Maillard reaction, the polyol pathway, and the glycoside pathway. Mechanisms of AGE formation inhibition by hydroxyl radical scavengers and superoxide radicals reduce oxidative stress, the formation of groups, and reactive dicarbonyl, inhibit carbonyl and dicarbonyl groups to reduce sugar and to reduce the Amadori products from the Maillard reaction, break the cross bond formed during AGE formation, and block RAGE activation. The blocking of AGE from binding to RAGE could inhibit the activation of the double signaling pathway to suppress ROS production [40,41].

## Conclusion

These results suggest that the combination of BST and PSP could be used to decrease AGE and RAGE expression and to improve spermatozoa quality. The combination of BST and PSP can be a promising candidate to develop as a food product to improve the spermatozoa quality in DM patients. Further research is needed before the combination could be applied to humans and to give an appropriate dose recommendation.

## Authors’ Contributions

AG and SRL conceptualized and designed the research. AG, AW, SNA, MFA, YA, and SRL conducted experiments, collected data, analyzed, and interpreted data. AG, AW, SNA, MFA, YA, and SRL wrote and revised the manuscript. AG obtained research funding and provided research material. AG, AW, SNA, MFA, YA, and SRL read and approved the final manuscript.
